# Parafoveal Processing in Chinese Sentence Reading: Early Extraction of Radical Level Phonology

**DOI:** 10.3389/fpsyg.2018.01605

**Published:** 2018-08-29

**Authors:** Jiefei Luo, Yan Wu, Runkai Jiao

**Affiliations:** School of Psychology, Northeast Normal University, Changchun, China

**Keywords:** Chinese character, phonetic radicals, phonogram regularity, sentence reading, preview benefit

## Abstract

The present study separated radical level phonology from character level phonology to explore the reliance on phonology during Chinese sentence reading with eye movement recording in a boundary paradigm. Participants viewed sentences with either regular, irregular, orthographically dissimilar homophone, or orthographically dissimilar non-homophone previews for the targets. Both regular and irregular characters contained the target character as the phonetic radical, with the regular character sharing the identical sound with its target phonetic radical. In Experiment 1, the irregular previews were different from the target phonetic radicals both in the first consonant and final compound vowels. In Experiment 2, the irregular characters would be replaced by the semi-regular previews, which shared the same final compound vowels but not the first consonant with the target characters. The radical level phonological preview benefit was obtained by the comparison between regular and irregular characters, while the character level phonological preview benefit was shown by the visually dissimilar homophones compared with the unrelated control condition. The preview benefit from parafoveal regular characters compared with irregular characters was observed in the first fixation duration, suggesting the early activation of phonological codes at radical levels. However, this preview benefit depends on phonological overlapping between the phonetic radicals and their host characters; it could be activated only when the pronunciation of the phonogram was totally consistent with that of its phonetic radical. Furthermore, the null preview effect of visually dissimilar homophones indicates no activation of phonological codes at the character level during Chinese sentence reading.

## Introduction

Phonology is the crucial information in a word’s formation. In alphabetic language, a great deal of research has proved the early role of phonology in activating the meaning of the visual word (Perfetti and Bell, 1991; Ferrand and Grainger, 1993; Ashby and Martin, 2008). However, in reading Chinese, whether phonological codes can be activated and then affect current processing is still an open question (Tan and Perfetti, 1998; Spinks et al., 2000; Tsai et al., 2004; Hsu et al., 2009). Chinese is characterized as an ideographic writing system. Unlike alphabetic language such as English, in which words are comprised of letters which represent phonemes, Chinese characters represent morphemic units and are composed of strokes and radicals in a square-shaped spatial configuration, which cannot be combined to represent the character’s sound. Chinese is considered as a writing system with deep orthography. The orthography-to-phonology mapping in Chinese is considered more opaque than in writing systems with shallow orthographies, such as English, Spanish, etc. Given the deep orthography of Chinese, some researchers assumed that in character reading, phonological information can be bypassed and semantic information is usually accessed directly from orthography (Wong and Chen, 1999; Meng et al., 2008; Zhou et al., 2018).

For instance, Wong and Chen (1999) first explored the effects of phonological information on Chinese sentence reading with eye movement recording in the violation paradigm. The basic manipulation was to change a critical character in a short passage, so that various combinations of orthographic and phonological information were altered. To illustrate, a critical Chinese character 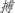 (*/bok3/* in Cantonese) could be replaced by an orthographically similar and phonologically identical character 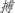 *(/bok3/)*, an orthographically similar but phonologically different character 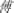 *(/foo6/)*, a homophone character without orthographic similarity 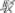 *(/bok3/)*, or an unrelated control character 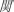 *(/gaan1/)*. Patterns of disruption caused by different manipulations were compared to reveal the use of orthographic and phonological information from individual characters. Results showed that orthographic manipulations produced reliable and early disruption in first fixation duration (FFD) in the target word position. FFD refers to the time that the eyes initially fixate on the target word independent of the number of fixations (Altarriba et al., 1996). It is reflecting the initial processing stage. In contrast, homophone effects were only found in the measure of a relatively late stage of processing (i.e., total reading time) in the target position but not in early measures of processing. Following this violation paradigm, Meng et al. (2008) also came to a similar conclusion, showing that Chinese readers relied more on orthographic information than on phonological information to access lexical semantics during sentence reading comprehension. This view is also called direct access hypothesis (Barron, 1986; Chen and Shu, 2001).

However, this view has been challenged by some researchers when the properties of Chinese phonograms were considered. In Chinese, more than 85% of all Chinese characters are phonograms; the composed phonetic radicals might be used to specify the pronunciation of the whole character (Lee et al., 2006a). For example, the phonetic radical “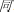, *tong2, same*” could show the pronunciation of its host character “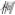, *tong2.*” A character is regular if the sound of the character is identical to that of its phonetic radical; otherwise, it is irregular. Pollatsek et al. (2000) noted that character regularity might confound the phonological benefits during character identity. When the target is an irregular character and the sound of its phonetic radical is activated, the competition between radical and character pronunciation could obscure the phonological effects. They manipulated the regularity and showed the naming latency was longer for irregular targets than for regular targets.

Following this idea, Tsai et al. (2004) separate regular from irregular characters to investigate radical level phonological activation. Homophone characters without orthographic similarity indicate character level phonological information. In this study, the boundary paradigm was used when eye movement was recorded. In this paradigm, a target location is identified in a sentence, and an invisible boundary is set just left of the target location (Rayner, 1975). When the reader starts to read the sentence, the preview word is presented at the target location, but it is replaced with the target as soon as the reader’s saccade crosses the boundary. If the preview word shares some properties with the target (such as the orthographic or phonological information), the viewing durations on the target word are shortened. This facilitation in viewing time is termed “preview benefit.” Results showed that when the previews were orthographically similar (sharing the same phonetic radicals) to the target, the data revealed a significant phonological benefit (regular vs. irregular characters) in FFD and gaze duration (GD). GD refers to the total amount of fixation time until the eyes leave the target word. It is usually interpreted as an index for a relatively later duration measure than FFD (Inhoff, 1984). When the previews were orthographically dissimilar to the target, the phonological benefit (homophone condition vs. unrelated control condition) in the FFD failed to approach significance; a benefit in GD was significant. Obviously, the phonological benefit from visually similar previews supports the earlier involvement of phonological computation at the radical level. This can be explained by the interactive activation model (Perfetti and Tan, 1998), which proposes that both orthographic and phonological information can cooperate in word identification. Actually, the importance of radicals in Chinese character reading has also been emphasized by Taft (2006) in the multilevel interaction activation model. In this model, a hierarchical activation framework is proposed, in which a visually presented Chinese character first activates visual feature unites representing strokes or stroke intersections, etc. Then the activating information activates the relevant radicals, which in turn sends activation information to the characters that contain those radicals. Therefore, the identification of a Chinese character involves the activation of radical relevant information.

However, interestingly, even with a similar design, Liu et al. (2002) came to a different conclusion. They found that a preview of the phonetic radical facilitated target reading, irrespective of the preview’s phonological similarity to the full target in FFD. Additionally, the orthographic preview benefit was shown in both FFD and GD, but the character level phonological preview benefit showed only in GD. They suggested that phonological coding might be slower or less important than orthographic coding in Chinese characters both at radical and character levels. Zhou et al. (2018) also supported this view by finding that preview effects of character level homophones were true only for children, not adults.

Obviously, even though the radical level phonological information was separated from that of the character level, researchers did not reach consensus on the issue of whether phonological codes could be activated earlier during Chinese character reading. However, an aspect deserving of mention is the fact that, in all prior studies, the target character was another phonogram different from the previews, which may be regular or irregular. For instance, Liu et al. (2002) reported that slightly more than half of the targets contained a phonetic radical with a similar pronunciation in their study. Lee et al. (2005) have proved that the reading speed of regular characters was faster than that of irregular ones. Therefore, radical level phonological activation may be confounded with the different identity speed of regular or irregular phonograms, inducing the inconsistency among different studies.

In addition, previous studies have found that Chinese individuals whose native language is Mandarin are more sensitive to perceiving final compound vowels than the first consonant. For instance, the interference from the final compound vowels to the first consonant was proved to be greater than that from the first consonant to the final compound vowels (e.g., Tong et al., 2008). Evidence from spoken character recognition also proved that sharing only the same final compound vowels can also facilitate character identification (e.g., Hu et al., 2012). Given the important role of vowels in Chinese character processing, the question as to whether phonological information could also be activated during sentence comprehension, if the phonograms shared only the same final compound vowels but not the first consonant with its critical phonetic radicals. This is interesting in that it can reveal to what extent the phonological code at the radical level could be activated in Chinese sentence processing.

Therefore, the present study separated radical level phonology from character level phonology to explore the reliance on phonology during the reading of sentences in a boundary paradigm. However, a simple character (e.g., 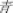 */qing1/, green*) instead of a phonogram was used as a target, so that the influence of target phonogram regularity on phonological activation at the radical level could be excluded. In Experiment 1, participants viewed sentences with either regular (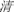 */qing1/, clean*), irregular (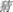 */cai1/, guess*), orthographically dissimilar homophone (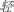 */qing1/, light*), or orthographically dissimilar non-homophone (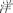 */yang1/, ocean*) previews for the targets. Both regular and irregular characters contained the target character as the phonetic radical, with the regular character sharing the identical sound with its target phonetic radical. In Experiment 1, the irregular previews were different from the target phonetic radicals both in the first consonant and final compound vowels. In Experiment 2, the irregular characters would be replaced by semi-regular previews, which share the same final compound vowels but not the first consonant with the target characters.

Since FFD is presumably the earliest measure of processing in reading, this dependent measure can be observed to reflect the role of phonology in early processing (Rayner, 1998). If the phonetic radical in the phonograms can be activated earlier regardless of phonological regularity, then it is expected that significant preview benefits from reading both regular and irregular characters in the FFD will be found and that there will be no significant difference between regular and irregular phonograms. In contrast, if phonogram regularity plays a significant role in the early stage of reading, only the regular phonogram can evoke the preview benefit, or the preview benefit from the regular phonograms will be greater than that of the irregular ones in the FFD. Namely, the FFD of regular phonograms will be shorter than in unrelated control conditions, and it will also be shorter than irregular characters. Besides, the setting of the homophone condition further verifies the phonological effects from the character level without orthographic similarity. According to previous research (e.g., Liu et al., 2002; Tsai et al., 2004), no preview benefit is expected, at least in the FFD. However, if the phonological overlapping between targets and previews influences the phonological activation at the radical level, results of semi-regulars will be different from those of irregulars.

## Experiment 1

### Methods

#### Participants

Twenty-four right-handed undergraduate students (eight males; mean age = 20.58; range 18–28) from Northeast Normal University took part in the experiment. All participants were native Chinese speakers with no history of psychiatric or neurological disorders, who had normal or corrected to normal vision. Each participant was required to sign the informed consent form before the experiment.

#### Design and Materials

First, 40 phonetic radicals that can stand alone as simple characters (such as *“
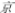
”*) were chosen as the critical characters, which were then used to form 40 two-character words. Among these two-character words, half of the critical characters appeared in the first position, whereas the other half appeared in the second position. Finally, 40 sentences containing these critical two-character words were formed (such as 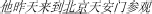 / *He visited Tiananmen Square in Beijing yesterday*). Each sentence’s range was 11–20 characters, with critical characters appearing in the middle of the sentence, approximately 5–10 characters from the left.

Nineteen undergraduate students who did not participate in the eye movement experiment were recruited to judge the familiarity of sentences (6-point Likert scale: 1-very familiar; 6-very unfamiliar). Results showed that these sentences were familiar to participants (*M* = 2.68, range 1.88–3.88). Four types of sentences were finally formed by changing the critical characters (such as 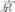, *jing1, the capital of a country*) into either a regular phonogram (e.g., 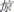, *jing1, scare*), an irregular phonogram (e.g., 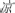, *liang2, cool*), a homophone character (e.g., 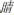, *jing1, eye*), or an unrelated control (e.g., 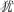, *you2, as if*).

All replaced phonograms had left-right structure, with semantic radical locating in the left position and phonetic radicals located in the right position. Character frequency (Modern Chinese Network Database^1^) and stroke number were matched among the four types of characters (*F*s < 1, *p*s > 0.5). The average frequency of critical two-character words (such as 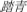) was 4.37 (*SD* = 4.77, values through log10 conversion). **Table 1** presents basic information about the stimuli.

**Table 1:** Example of characters, mean frequency, stroke number, and pronunciation of targets and previews.

		Critical simple character and its replaced phonograms			
	Simple character	Regular	Irregular	Homophone	Unrelated control
Example	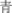	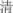	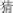	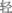	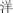
Stroke number	5.73 (2.31)	9.08 (2.76)	9.28 (2.80)	9.60 (2.35)	8.85 (2.34)
Frequency (log10)	5.16 (5.27)	4.42 (4.81)	4.56 (4.88)	4.56 (4.78)	4.53 (4.96)
Pronunciation	qing1	qing1	cai1	qing1	yang2

*The values in parentheses were the standard deviation, log10 refers to the average character frequency taken with 10 for the bottom of the logarithm.*

To avoid sentence repetition, four lists were constructed such that each target appeared only once on a list, and all possible sentences were exhausted across lists. Ten sentences were presented on a list per condition. To conceal the real purpose of the experiment from the participants, another 40 normal sentences, with similar content and length as the experimental sentences, were added as fillers (i.e., 80 sentences on a list). In addition, to ensure participants read the sentences carefully, 20 questions about the fillers were randomly presented to be answered. Participants were asked to judge whether the content presented was correct or not.

#### Apparatus

The EyeLink 1000 Plus tracker (SR Research Ltd.) was used to present the materials and record eye movement data. Although reading was binocular, eye movements were recorded only from the right eye, using chin rests to minimize head movements. Sentences were presented on the 20-inch Dell monitor with a sampling rate of 1000 Hz. Screen resolution was 1024 × 768 pixels and interfaced with a PC at a viewing distance of 80 cm, which ensured the visual angle of a single character was about 1^°^. Custom-designed software ensured that display change occurred within 6–12 ms. Sentences were presented in white, regular script, size 28 font on a black background. The eye movement trajectory of participants was recorded in the computer using a high-speed camera on the EyeLink tracker. Materials presentation and eye movement recording were conducted using dedicated software.

#### Procedure

**FIGURE 1:**
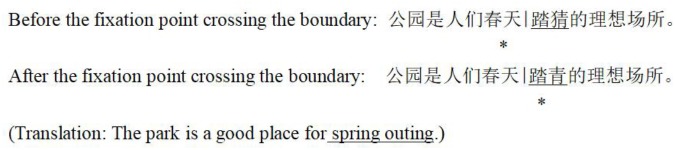
Schematic diagram of the boundary paradigm.

As shown in **Figure 1**, in the boundary paradigm, an invisible boundary (expressed by “—”) is set before the target two-character words (e.g., 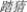). Before the fixation point (expressed by “^∗^”) crosses the boundary, a violation two-character word is presented in the target position. Once the fixation point crosses the boundary, the violation two-character words change into normal two-character words.

All participants were measured individually in a sound-proof room. Calibration, validation, and drift correct were conducted before the experiment to ensure the accuracy of the eye movement track record. In calibration, nine standard points (white dots) randomly appeared on the screen. Participants were asked to look at the points one by one until they disappeared. Validation followed calibration; nine standard points were again presented on the screen. The procedure and scale mark were the same as before. If both calibration and validation were successful, a drift correct was performed. A calibration point appeared on the screen and participants were asked to look at the point. When the drift correct was successful, sentence reading could begin. One sentence was presented on one screen. Participants were asked to press a corresponding key (i.e., “Space”) once they finished the reading. The whole experiment lasted approximately 20 min.

### Results and Analysis

First, all accuracy rates for the questions were more than 85%, indicating that participants read the sentences carefully. We took the target two-character word (N+1 area) after the boundary and a character before the boundary (N area) as the two interest areas. The eye movement indexes in the current research included the FFD, GD, and regression path reading time. Regression path reading time refers to the summed fixation times, starting when entering a region, until the region’s right boundary is crossed and including fixations to the left of the region, if any (Calvo, 2001). The first two indexes are assumed to reflect the earlier processing stage, in which the FFD is earlier than the GD, and the last is assumed to reflect the re-reading stage (Wong and Chen, 1999). The regression path reading time contains the reading time related to the regressive behavior, reflecting the process of detecting differences and re-reading the preceding text. In other words, this index not only reflects the processing of lexical access but also the process of sentence integration in the later period. The single fixation points that were shorter than 60 ms or longer than 800 ms (Cui et al., 2013) would be deleted. We also deleted the reading time of FFD and GD, and the regression path reading time that was above or below three standard deviations from the mean.

For each index, the Linear Mixed-effect Model (LMM; Baayen et al., 2008) was used, based on the R language environment (R Development Core Team, 2014). Lme4 data package (Bates et al., 2012) was performed with the character type (regular vs. irregular vs. homophone vs. unrelated control) as the fixed factor, and subjects and items as random factors.

Results of N area serve as evidence for the existence of the parafoveal-on-foveal effect (Kliegl et al., 2007). However, no significant effects were observed in this area for all indexes (*p*s > 0.1), which suggested that the information of the parafoveal did not affect the processing of the N area before the boundary. Results of N+1 area can reveal the preview effect on the early indicators of sentence processing. **Table 2** presents the mean reading time and standard deviation of the four conditions in each index. The first level of analysis, using the simple effect model, compared the regular, irregular, and homophone conditions with the unrelated control condition.

**Table 2:** Mean reading time (ms) and standard deviation in the four conditions.

	FFD		GD		Regression path reading time	
	M	SD	M	SD	M	SD
Regular	248	105	399	210	525	242
Irregular	274	115	412	216	563	253
Homophone	274	115	457	226	591	266
Unrelated control	286	119	458	217	587	249

#### FFD

LMM analysis showed that the first FFD of regular phonograms was shorter than that of the unrelated control condition (*b* = 37.408, *SE* = 10.142, *t* = 3.688, *p* < 0.001). There was no significant difference between the other two comparisons (irregular phonograms or homophones vs. unrelated control) (*p*s > 0.1).

#### GD

LMM analysis revealed that the GD of the regular phonograms (*b* = 59.18, *SE* = 18.13, *t* = 3.265, *p* < 0.01) and irregular phonograms (*b* = 45.74, *SE* = 18.04, *t* = 2.535, *p* < 0.05) were slower than that of unrelated control conditions, but there was no significant difference between homophones and the unrelated control condition (*p* > 0.4).

#### Regression Path Reading Time

Regular phonograms showed a shorter regression path reading time than the unrelated control condition (*b* = 61.07, *SE* = 20.59, *t* = 2.966, *p* < 0.01), but no significant differences between irregular phonograms and unrelated control condition or between homophones and unrelated control condition were found (*p*s > 0.1).

In the second level of analysis, a linear model was established to further examine the differences between any of the other two conditions (i.e., regular vs. irregular; regular vs. homophone; irregular vs. homophone). LMM analysis revealed a significant difference between the regular and irregular phonograms (*b* = 26.191, *SE* = 10.178, *t* = 2.573, *p* < 0.05), or between the regular phonograms and homophones (*b* = 38.134, *SE* = 10.234, *t* = 3.726, *p* < 0.001) for the FFD, but the difference between the irregular phonograms and homophones was not significant (*p* > 0.1).

When the GD was taken into account, the difference between the regular and irregular phonograms was no more significant (*b* = 13.439, *SE* = 18.192, *t* = 0.739, *p* = 0.46). In contrast, the difference between the irregular condition and homophones became significant (*b* = 44.70, *SE* = 18.21, *t* = 2.455, *p* < 0.05), and the GD of regular phonograms was again shorter than that of homophones (*b* = 58.14, *SE* = 18.29, *t* = 3.178, *p* < 0.01). For the regression path reading time, the differences between the regular and irregular conditions or between the irregular and homophone conditions were not significant any more. The regular phonograms showed shorter regression path reading time than the homophone condition (*b* = 66.01, *SE* = 20.78, *t* = 3.177, *p* < 0.01).

To summarize, the preview benefit of regular characters was significant in the FFD, GD, and regression path reading time, while the preview benefit of irregular characters failed to approach significance in the FFD; the benefits in GD and regression path reading time were significant. The difference between regular and irregular previews was only significant in FFD but not in GD and go path reading time. However, no significant preview effect was found when the preview was an orthographically dissimilar homophone. The results showed that at the initial processing stage, the phonological information at the radical level was activated to promote the preview benefit. However, at the later stage, the orthographic information of the phonetic radical again became the dominant information to facilitate character identification.

In Experiment 2, irregular phonograms would be replaced by semi-regular characters, in which Chinese phonograms shared the same final compound vowels but had different first consonants from the critical phonetic radicals. The other conditions were kept the same as Experiment 1. If phonological information of semi-regular phonograms could also be activated during the early stage of sentence processing, we would find a significant difference between the semi-regular phonograms and the unrelated control condition in FFD. Also, the difference between the semi-regular and regular phonograms would not be significant any more.

## Experiment 2

### Methods

#### Participants

Thirty-two right-handed undergraduate students (nine males; mean age = 20.69; range 18–27) from Northeast Normal University took part in the experiment. All participants were native Chinese speakers, with no history of psychiatric or neurological disorders, and had normal or corrected to normal vision.

#### Design and Materials

Like Experiment 1, there were four types of replaced characters: regular, semi-regular, homophone and unrelated control characters. Semi-regular characters also contained the critical characters as phonetic radicals and shared the same final compound vowels but had a different first consonant. Again, stroke number and character frequency (see **Table 3**) were matched among the four conditions (*F*s < 1, *p*s > 0.5), and the average frequency of critical two-character words was 4.39 (*SD* = 4.71, values through log10 conversion). All experimental materials were familiar to undergraduate students, since the average rating of familiarity was 2.66 (range 1.65–4.1; 6-point Likert scale: 1-very familiar; 6-very unfamiliar). All other conditions were the same as in Experiment 1, including apparatus and procedure.

**Table 3:** Example of characters, mean frequency, stroke number, and pronunciation of targets and previews.

	Critical simple character and its replaced phonograms				
	Simple Character	Regular	Semi-regular	Homophone	Unrelated control
Example	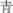	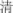	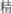	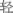	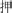
Stroke number	5.73 (1.97)	9.28 (2.35)	9.00 (2.26)	9.35 (2.65)	9.08 (2.40)
Frequency(log10)	5.16 (5.36)	4.40 (4.51)	4.42 (4.58)	4.58 (4.81)	4.37 (4.44)
Pronunciation	qing1	qing1	jing1	qing1	ya1

*The values in parentheses were the standard deviation, log10 refers to the average character frequency taken with 10 for the bottom of the logarithm.*

### Results and Analysis

All accuracy rates for participants answering the questions were more than 90%, indicating participants read the sentences carefully. Again, the target two-character word (N+1 area) and a character before it (N area) were taken as the interest areas. Single fixation points shorter than 60 ms or longer than 800 ms, as well as reading time larger than 3*SD* (2.75%) were excluded from the final analysis. Similarly, the LMM based on the R language environment was used to analyze the data. The same two levels of analysis as Experiment 1 were conducted to reveal the effects of phonogram regularity in this experiment.

At the two levels of analysis, no significant effects were found in the N area (*p*s > 0.1), but in the N+1 area, we did find some significant effects in FFD, GD, and regression path reading time. **Table 4** presents the mean reading time and standard deviation of the four conditions for each index of this area.

**Table 4:** Mean reading time (ms) and standard deviation in the four conditions.

	FFD		GD		Regression path reading time	
	M	SD	M	SD	M	SD
Regular	248	118	370	203	440	219
Semi-regular	268	132	374	202	454	226
Homophone	270	129	399	198	477	226
Unrelated control	283	130	412	200	496	211

At the first level of analysis, for the FFD, only the difference between regular phonograms and the unrelated control condition was significant (*b* = 34.199, *SE* = 9.859, *t* = 3.469, *p* < 0.001). The other two comparisons (semi-regular vs. unrelated control; homophone vs. unrelated control) were not significant (*p*s > 0.1). For the GD, both regular phonograms (*b* = 41.48, *SE* = 13.79, *t* = 3.008, *p* < 0.01) and semi-regular characters (*b* = 37.08, *SE* = 13.82, *t* = 2.683, *p* < 0.01) showed shorter reading time than the unrelated control condition. However, there was no significant difference between homophone phonograms and the unrelated control condition (*p*s > 0.1). For the regression path reading time, again, as compared with the unrelated control condition, both regular phonograms (*b* = 54.98, *SE* = 14.87, *t* = 3.697, *p* < 0.001) and semi-regular phonograms (*b* = 39.92, *SE* = 14.91, *t* = 2.678, *p* < 0.01) showed shorter reading time. No significant difference between homophone phonograms and the unrelated control condition was found (*p*s > 0.1).

At the second level, the FFD of regular phonograms was shorter than that of semi-regular phonograms (*b* = 19.266, *SE* = 9.866, *t* = 1.953, *p* = 0.05) and homophone characters (*b* = 20.800, *SE* = 9.785, *t* = 2.216, *p* < 0.05). For the GD, the difference between regular and homophone phonograms was significant (*b* = 28.52, *SE* = 13.68, *t* = 2.084, *p* < 0.05), and the difference between semi-regular and homophone phonograms was marginally significant (*b* = 24.12, *SE* = 13.72, *t* = 1.758, *p* = 0.079). For the regression path reading time, analysis again revealed the significant difference between regular and homophone phonograms (*b* = 33.77, *SE* = 14.75, *t* = 2.289, *p* < 0.05). Apart from this, all the other effects were not significant.

Again, parafoveally presented regular characters facilitated the processing of the target character in FFD, and the difference between regular and semi-regular conditions reached significant. However, no preview benefit was obtained from semi-regular previews in FFD, relative to the unrelated control condition. These results indicate that just sharing final compound vowels is not enough to evoke phonetic radicals during the initial stage of character processing.

## Discussion

The present study separated radical level phonology from character level phonology to explore the reliance on phonology during silent reading of Chinese sentences. The radical level phonological preview is shown by the comparison between regular and irregular characters, while the character level phonological preview benefit is shown by the visually dissimilar homophones compared with the unrelated control. The novel contribution of the present study is the fact that we found a preview benefit of phonological codes at the radical level in the FFD but not at the character level.

As shown by results, parafoveally presented regular previews facilitated processing of the target character in FFD, relative to unrelated control preview characters. The benefit was not due to the whole character level phonological similarity between previews and targets, because the difference between regular and homophone conditions was significant in FFD. Additionally, the benefit was not solely due to the activation of phonetic radicals in preview characters, because the difference between regular and irregular conditions was also significant in FFD. Therefore, it was the phonological similarity between phonetic radicals and host characters that finally facilitated the activation of target phonetic radicals in the initial processing period. The results are in line with the findings of Tsai et al. (2004), who also found significant facilitation effects of phonogram regularity during the initial processing stage, as reflected by the FFD. However, beyond the findings of Tsai et al. (2004), we found that the phonology of phonetic radicals could be evoked to facilitate current processing only when the pronunciation of phonetic radicals was exactly the same as that of its host characters.

Actually, the insignificant preview effects of irregular or semi-regular preview characters in FFD could also be assumed as evidence of the earlier activation of radical level phonology. In irregular/semi-regular characters, the sound of their phonetic radicals is also activated, and null preview benefits of phonetic radicals in FFD are simply due to the competition between radical and character pronunciation. If this interpretation makes sense, when the orthographic similarity between previews and targets was obtained through the character levels without sharing the same phonetic radicals (e.g.: 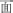
*surface* vs. 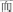
*while*), the orthographic information could exert earlier parafoveal preview effects which is exactly what was observed in the literature. When the target was a simple character, the visually similar preview facilitated current processing by showing the significant recovery effects in the violation paradigm (Meng et al., 2008; Liu et al., 2011) and preview effects in the boundary paradigm in FFD (Liu et al., 2002; Zhou et al., 2018).

The early activation of phonology at the radical level could be owing to the function role of the phonetic radical in a phonogram. Several studies have probed the issue of radical level phonological processing in reading Chinese using either indexes of regularity or indexes of consistency to describe the mappings between Chinese orthography and phonology (Seidenberg, 1985; Hue, 1992; Lee et al., 2005, 2006b; Hsu et al., 2009). Consistency refers to whether a character’s pronunciation agrees with that of its orthographic neighbors that contain the same phonetic radical. Behaviorally, the naming speed for irregular or inconsistent characters is found to be much slower than for regular or consistent characters, especially for low-frequency characters. This means the frequency was interacted with character regularity/consistency, which then indicates Chinese phonograms are not read via a direct association between orthography and phonology but through the process of sublexical phonology (Seidenberg, 1985; Fang et al., 1986; Hue, 1992). Agreeing with this argument, we found significant radical level phonological activation in Chinese sentence reading. Additionally, the important role of phonetic radicals in Chinese character reading is compatible with the view of multilevel interaction activation model (Taft, 2006). In this model, a visually presented Chinese character is assumed to be activated by the radical level representation. Thus, as an important type of Chinese radical, information related to phonetic radicals are expected to be activated in recognizing a Chinese character.

Nevertheless, note that current results also indicate that regularity effects happen only when the sound of a character is totally identical to that of its phonetic radical. Sharing the final compound vowels was not enough to facilitate the activation of phonetic radicals in the initial stage of Chinese character reading. This can be shown by the null preview benefit of the semi-regular character, relative to unrelated control conditions in FFD. However, prior research has indeed found the important role of vowels during Chinese character processing, especially in spoken character recognition. For instance, using event-related potentials, Hu et al. (2012) measured the access of suprasegmental (tone) and segmental (vowel) information in spoken character recognition with Mandarin idioms. Participants performed a delayed-response acceptability task, in which they judged the correctness of the last word of each idiom, which might deviate from the correct word in either tone or vowel. Results showed that, compared with the correct idioms, a larger early negativity appeared only for vowel violation. Additionally, a larger N400 effect was observed for vowel mismatch than tone mismatch. Using a speeded classification paradigm, Tong et al. (2008) examined processing interactions between segmental (consonant, vowel) and suprasegmental (tone) dimensions of Mandarin Chinese. Results showed that vowels exerted greater interference on consonants and tones than the consonants and tones exerting on vowels. These results suggest that Chinese listeners are more sensitive to a change in vowels, which then evokes a predominant role overriding the tone and constant in spoken character recognition. In contrast, current findings reveal that Chinese readers are not as sensitive to vowels. One possible reason for this inconsistency may lie in the different mechanisms of visual character identification and spoken character recognition, with the former one relying less on phonologically related information.

Surprisingly, although phonogram regularity plays an early role in Chinese sentence reading, this facilitation effect finally disappears as processing progresses. For example, the differences between regular and irregular characters or between regular and semi-regular characters became insignificant in the GD and go path reading time, but all three conditions were significantly different from the unrelated control conditions. This indicates that the phonology of the phonetic radical no longer works, and its orthography ultimately plays a key role during the re-processing stage. The result is also different from that of Tsai et al. (2004), in which the preview benefit of regularity approached significance in both the FFD and GD. One possible reason for the inconsistency is the fact that in the current study, the target character was a simple character but not a phonogram. In Chinese, less than 10% of characters are simple characters, and most of them are learned during the first two grades of elementary school (Shu et al., 2003). Relative to the compound characters, simple characters have higher familiarity and frequency. Sentence reading studies with English texts have shown that phonology effects tend to be relatively small when high-frequency words are encountered (e.g., Jared et al., 1999), presumably because these words are relatively easy to identify. The relatively high predictability of target characters could have had the effect of making their recognition also relatively easy, thus diminishing the size and robustness of radical level phonology benefits.

In addition, we didn’t find a significant preview benefit from the visual dissimilar homophone characters. As previously stated, homophone preview effects indicate character level phonological preprocessing. Although remnants of inner voice can still be found in reading among Chinese readers (e.g., Yan et al., 2014), it is commonly accepted that pure parafoveal homophone effects without orthographic similarity during Chinese sentence reading may not be as effective. First, homophonic preview effects have been consistently shown only for GD during Chinese sentence reading (Liu et al., 2002; Tsai et al., 2004; Yan et al., 2009). Arguably, as GD includes re-fixations, it is usually interpreted as an index for a relatively later duration measure than FFD (Inhoff, 1984). Second, effective parafoveal phonological extraction is highly restricted to favorable situations including long preview durations (i.e., fixation duration on pretarget words) and high parafoveal processing efficiency afforded by high-frequency pretarget words (Tsai et al., 2012). Finally, recent evidence suggests that homophonic preview effects are limited to oral reading but not silent reading, because the explicit demand of phonological processing in oral reading increases the parafoveal processing of phonology (Pan et al., 2016). Given the unstable effects of character level phonology, it is not surprising that the current study did not reveal significant preview effects of visually dissimilar homophones among Chinese readers. In fact, it has been well documented that the orthographic depth of the writing system has an influence on phonological processing. For instance, English readers were found to rely less on phonology as compared to Spanish, German, or French readers (Sprenger-Charolles et al., 2003), since grapheme-phoneme rules in English are not as regular as the other three languages. This may suggest a trivial role of phonology among Chinese readers, because the lexical level orthography-to-phonology mapping is quite opaque in Chinese.

Traditionally, direct access hypotheses assumes that the semantic meaning of a visual word is activated by orthographic code directly, and it bypasses the phonological information (Barron, 1986; Chen and Shu, 2001), which means sub-lexical processing can be directly transformed from orthographic to semantic units. By contrast, the model holding on interactive activation view proposes that different linguistic codes, including orthography and phonology interact with each other to identify a visually presented character (Perfetti and Tan, 1998). Obviously, current findings support the view of interactive activation by confirming that both orthographic and phonological codes contributing to character identification. However, in Chinese, characters are not read via a direct association between orthography and phonology but through the process of sublexical phonology. When the sound of a character is identical to that of its phonetic radical, the sublexical phonology facilitates the orthographic retrieval of Chinese characters, but when their sounds are different from each other, the competition between sublexical phonology and their host character will inhibit the orthographic activation of the target character.

## Conclusion

To summarize, in the present study, we extend our current understanding of the reliance on phonology from parafoveal processing in Chinese; that is, how various types of phonological information are processed prior to a word being fixated. The preview effect from parafoveal regular characters compared with irregular characters was observed in FTD during the reading of Chinese sentences, suggesting the early activation of phonological codes at radical levels. However, this preview benefit depends on phonological overlapping between phonetic radicals and their host characters, and can be activated only when the pronunciation of phonograms is identical to that of their phonetic radicals. Furthermore, the null preview effect of visually dissimilar homophones indicates no activation of phonological codes at the character level during Chinese sentence reading.

## Ethics Statement

This study was carried out in accordance with the recommendations of “Ethical Issues and Body Protection Guidelines in Psychology, the Ethics Committee of School of Psychology at Northeast Normal University” with written informed consent from all subjects. All subjects gave written informed consent in accordance with the Declaration of Helsinki. The protocol was approved by the “The Ethics Committee of School of Psychology at Northeast Normal University.”

## Author Contributions

JL and YW contributed to the conception and design of the work. JL collected and analyzed the data. All authors contributed to discussion and writing of the manuscript.

## Conflict of Interest Statement

The authors declare that the research was conducted in the absence of any commercial or financial relationships that could be construed as a potential conflict of interest.

## Footnotes

1 http://lingua.mtsu.edu/chinese-computing/
